# AI-2 quorum sensing-induced galactose metabolism activation in *Streptococcus suis* enhances capsular polysaccharide-associated virulence

**DOI:** 10.1186/s13567-024-01335-5

**Published:** 2024-06-17

**Authors:** Shuji Gao, Chenlong Mao, Shuo Yuan, Yingying Quan, Wenjie Jin, Yamin Shen, Xiaoling Zhang, Yuxin Wang, Li Yi, Yang Wang

**Affiliations:** 1 College of Animal Science and Technology, Henan University of Science and Technology, Luoyang, 471000 China; 2 College of Life Science, Luoyang Normal University, Luoyang, 471934 China; 3 Henan Provincial Engineering Research Center for Detection and Prevention and Control of Emerging Infectious Diseases in Livestock and Poultry, Luoyang, 471003 China

**Keywords:** *Streptococcus suis*, galactose metabolism, AI-2 quorum sensing, capsular polysaccharide, virulence

## Abstract

Bacteria utilize intercellular communication to orchestrate essential cellular processes, adapt to environmental changes, develop antibiotic tolerance, and enhance virulence. This communication, known as quorum sensing (QS), is mediated by the exchange of small signalling molecules called autoinducers. AI-2 QS, regulated by the metabolic enzyme LuxS (S-ribosylhomocysteine lyase), acts as a universal intercellular communication mechanism across gram-positive and gram-negative bacteria and is crucial for diverse bacterial processes. In this study, we demonstrated that in *Streptococcus suis* (*S. suis*), a notable zoonotic pathogen, AI-2 QS enhances galactose utilization, upregulates the Leloir pathway for capsular polysaccharide (CPS) precursor production, and boosts CPS synthesis, leading to increased resistance to macrophage phagocytosis. Additionally, our molecular docking and dynamics simulations suggest that, similar to *S. pneumoniae*, FruA, a fructose-specific phosphoenolpyruvate phosphotransferase system prevalent in gram-positive pathogens, may also function as an AI-2 membrane surface receptor in *S. suis*. In conclusion, our study demonstrated the significance of AI-2 in the synthesis of galactose metabolism-dependent CPS in *S. suis*. Additionally, we conducted a preliminary analysis of the potential role of FruA as a membrane surface receptor for *S. suis* AI-2.

**Supplementary Information:**

The online version contains supplementary material available at 10.1186/s13567-024-01335-5.

## Introduction

*Streptococcus suis* (*S. suis*), a zoonotic gram-positive bacterium, is part of the normal microbial flora in the upper respiratory tract of swine. Additionally, *S. suis* is a significant human pathogen that causes diseases such as meningitis and toxic shock-like syndrome following infection via wounds or the respiratory tract [1]. Since its initial documentation as a cause of human infection in Denmark in 1968, global research interest in *S. suis* has consistently increased. Notable public health incidents involving human infections with *S. suis* serotype 2 occurred in China in 1998 and 2005. These incidents are characterized by streptococcal toxic shock syndrome (STSS), resulting in a grim prognosis and high mortality [2].

The survival of *S. suis* in the respiratory tract, a key factor in its transmission and infection, is primarily associated with its virulence factor, capsular polysaccharide (CPS) [3]. CPS plays a role in adhesion and is a crucial virulence factor facilitating bacterial invasion of host cells [4, 5]. In 2001, Magee and Yother highlighted the role of CPS in *Streptococcus pneumoniae* (*S. pneumoniae*) colonization, noting that mutants producing only 20% CPS exhibited reduced invasive and lethal capabilities [6]. Deletion of *frwC* (encoding hypothetical fructose-specific enzyme II C) in *Klebsiella pneumoniae* was found to enhance *magA* (encoding a polymerase essential for capsule synthesis) transcription, thereby increasing CPS synthesis [7]. In group B streptococci (GBS), the Δ*cpsE* mutant was shown to secrete fewer carbohydrates than the wild-type strain (WT), leading to a reduction in CPS synthesis. Concurrently, the Δ*cpsE* mutant showed increased susceptibility to ingestion by human placental macrophages [8]. Furthermore, CPSs are instrumental in bacterial niche adaptation, drug resistance evolution and penetration of the blood‒brain barrier [9–11].

Quorum sensing (QS) represents a cell-to-cell communication process occurring both within and among bacterial species [12]. QS can be categorized into three major groups: (i) LuxI/R-type QS in gram-negative bacteria, (ii) oligopeptide two-component QS in gram-positive bacteria, and (iii) the interspecies communication system known as LuxS-mediated AI-2 QS found in various microbial communities [13]. Our research has established the global regulatory impact of AI-2 QS on *S. suis* virulence [14–16], although the specific regulatory pathway involved remains elusive. Ongoing analysis of QS regulatory mechanisms has revealed a connection to CPS production. For instance, rgg/shp QS is involved in the synthesis of the CPS matrix hyaluronic acid in *Streptococcus zooepidemicus*. Both Δ*shp* and Δ*rgg-shp* exhibited reduced CPS synthesis and impaired biofilm formation [17]. Zhi et al. confirmed Rgg/Shp144 and Rgg/Shp939 as additional QS agents in *S. pneumoniae*. Microarray analysis revealed that Rgg939 regulates the SPD_0940-SPD_0949 locus, with *mnaA-* and *mnaB*-encoded proteins participating in the synthesis of N-acetylmannosaminuronic acid (UDP-ManNAcA), a component of the serotypes 12F and 12A CPS [18]. It is known that in gram-negative bacteria, AI-2 uptake is mediated by the Lsr transporter and is subsequently phosphorylated. Phosphorylated AI-2 cannot traverse the cell membrane, leading to the accumulation of AI-2-P, ultimately triggering a density-dependent cellular response [19, 20]. However, a significant knowledge gap exists in the understanding of AI-2 transport within gram-positive bacteria. AI-2 is fundamentally a pentose sugar with two ketone groups. Low-G + C gram-positive bacteria exhibit a substantial presence of carbohydrate transporters on their cell surface. These proteins are prime candidates for potentially serving as AI-2 transporters, in addition to their role in binding extracellular polysaccharides [21]. To date, the sole reported AI-2 receptor among gram-positive bacteria is *S. pneumoniae*. Trappetti et al. demonstrated that AI-2 enhances *S. pneumoniae*’s ability to use galactose as a carbon source and upregulates the Leloir pathway through the phosphotransferase FruA, impacting CPS production and increasing toxicity [22]. However, the specific mechanism by which AI-2 QS regulates the CPS in *S. suis* remains uncertain. CPS is pivotal for host invasion and constitutes a fundamental aspect of *S. suis* pathogenesis. Consequently, further exploration of the regulatory pathways of *S. suis* CPS is imperative. This study investigated the potential interplay between AI-2 QS and CPS synthesis in *S. suis* from a bacterial metabolic perspective and preliminarily evaluated the potential of FruA as a membrane surface receptor for *S. suis* that senses AI-2 signalling molecules to regulate the Leloir pathway, aiming to provide a theoretical foundation for the prevention and control of *S. suis* infection.

## Materials and methods

### Bacterial strains and growth conditions

The *S. suis* wild-type (WT) and Δ*luxS* (*luxS* gene mutant) strains were constructed previously [23]. Capsule quantification, metabolomic analysis, and metabolic experiments were carried out using cells cultivated in chemically defined medium (CDM). Glucose (Glc) and galactose (Gal) served as the respective carbon sources [24, 25]. In animal challenge experiments, *S. suis* was cultured in nutrient broth enriched with 10% (v/v) fetal bovine serum. The AI-2 precursor molecule DPD was obtained from Omm Scientific, Inc., TX. Unless otherwise specified, the experimental concentration used was 4 μM [26].

### Growth assays

The assessment of the growth kinetics of *S. suis* was conducted using the established methodology outlined by Li et al. [16]. Briefly, the bacterial cultures were incubated at 37 °C. Subsequently, the samples were diluted and plated on TSB agar for colony-forming unit (CFU) enumeration.

### Capsular polysaccharide formation assay

*S. suis* was grown in CDM-Gal, and CPS was extracted and quantified following the protocol detailed by Li et al. [16]. The sample was prepared to achieve a solution concentration of 5 mg/mL. The optical density at 490 nm was measured and substituted into the standard curve equation to calculate the CPS content, as detailed in Additional file 1.

### PMP–HPLC analysis

According to previous methods and with slight adjustments [27], high-performance liquid chromatography (HPLC) (Waters, Shanghai, China) was used to analyse the effect of AI-2 QS on the composition of *S. suis* CPS. The chromatographic conditions were as follows: chromatographic column: Waters XBridge C18 column 5 μm, 4.6 × 250 mm (Waters, Shanghai, China); mobile phase: phosphate buffer (pH 6.7)/CH_3_CN (80:20); flow rate: 1 mL/min; column temperature: 30 ℃; injection volume: 20 μL; and detector: UV detector (245 nm). The proportion of each monosaccharide component was obtained according to the peak area of each monosaccharide component.

### Transmission electron microscopy (TEM)

*S. suis* (10^6^ CFU/mL) grown in CDM-Gal was fixed in 2.5% glutaraldehyde (1 mL). Then, the cells were treated with 2% osmium tetroxide for 2 h. After dehydration, the cells were embedded in epoxy resin, and their morphology was evaluated using an H-7650 transmission electron microscope (Hitachi, Tokyo, Japan) [28].

### Anti-phagocytosis assay

Following a slightly modified methodology from Hui et al. [29], the RAW264.7 murine macrophage cell line was used to assess the antiphagocytic properties of *S. suis*. RAW264.7 cells were cultured overnight in DMEM supplemented with 10% fetal bovine serum (FBS) at 37 °C in a 5% CO_2_ atmosphere to form monolayers, and 10^5^ cells were seeded per well in a 24-well plate. *S. suis* (10^6^ CFU/mL) was added to infect the cells for 2 h, 4 h, or 8 h. Subsequently, DMEM supplemented with 0.1 mg/mL penicillin and streptomycin was added to the wells. After 2 h of incubation, the cells were lysed using an ice-water mixture, followed by serial dilution and plating onto TSB agar. CFU were quantified after a 24-h incubation at 37 °C.

### Metabolomic analysis based on LC‒MS/MS

The impact of AI-2 on the metabolome of *S. suis* was assessed following a method based on Husna et al. [30, 31], with some adjustments. *S. suis* was cultured in CDM-Gal for 8 h and rapidly quenched with liquid nitrogen. Subsequently, the bacterial cells were combined, and ultrasonic crushing was carried out in a cold extract (methanol:acetonitrile:water, 2:2:1). After centrifugation for 15 min at 4 °C and 12 000 rpm, the supernatant was collected and analysed. Each experimental group included six biological replicates.

Untargeted metabolite profiling was performed utilizing a Thermo-Fisher UPLC system (Thermo-Fisher, San Jose, CA, USA) coupled with an LTQ XL mass spectrometer. Mobile phase B consisted of methanol (HPLC grade, Merck, Germany) with a flow rate of 0.3 mL/min. An injection of 2 µL of sample was made, and the gradient profile proceeded as follows: 0–8 min (5% B), 8–18 min (35% B), 18–22 min (35% B), 22–28 min (90% B), 28–30 min (90% B), and 30–32 min (50% B). For mass spectrometry, the conditions were configured as follows: an ESI ion source with a spray voltage of 3700 V (positive ion mode) or −3000 V (negative ion mode), a scanning range spanning from 65 to 995 m/z, a first-level resolution of 70 000 and a secondary resolution of 17 500. Collision energy was applied incrementally with values of 3, 20 eV, 40 eV, and 60 eV. The scanning rate was set at 7 Hz.

### Quantitative RT‒PCR (qRT‒PCR)

Total RNA was extracted via the TRIzol method, and cDNA was synthesized via reverse transcription following the protocol described by Wang et al. [32]. Specific primers targeting 16S rRNA were used as internal controls. The amplification data were subjected to analysis using the comparative critical threshold (2^−ΔΔCΤ^) method and are expressed as the overall expression relative to that of the 16S rRNA. Comprehensive primer details can be found in Table 1.

**Table 1:** **Primers used for the quantitative RT‒PCR analysis**.

Genes	Primer sequence
16S rRNA-1	GTTGCGAACGGGTGAGTAA
16S rRNA*-*2	TCTCAGGTCGGCTATGTATCG
*galR*-1	CGGTCAGCGTATCAGAGTCC
*galR*-2	CTCCAAGAAGTGGACGGCAT
*galK*-1	AGGCGGATAACTGGACCAAC
*galK*-2	ACAAGCCTGAACCGTTTGGA
*galT*-1	CGGGTGGCTCTATCTTGACC
*galT*-2	GGGCCATTTGACAATGCCAG
*galE*-1	TGGGCACCGATTGGATTGAA
*galE*-2	TATGCCAACTAGCGCAACGA
*cps2G*-1	AGGCTCTTGTCATTGGTATG
*cps2G*-2	TACTCGCCACTCTTCTCC
*cps2E*-1	CCATTACCGCTCTATTATTCTG
*cps2E*-2	GCCTACATCAATACCTAACAAC

### Virtual molecular docking assays

The three-dimensional (3D) structure of AI-2 was obtained from PubChem [33]. The protein sequences of FruA (QOE30179.1 and VDG78558.1) were retrieved from the NCBI database. The 3D structure of FruA was generated using the SWISS-MODEL website [34]. To assess quality, the Ramachandran diagram was generated through SAVES v6.0 [35], and the results were analysed by QMEAN [36, 37]. Virtual molecular docking experiments were conducted utilizing AutoDock 4 and AutoDock Vina, in accordance with the methodologies outlined by Joshi et al. [38] and Wang et al. [39]. The docking results were visualized using PyMOL.

### Molecular dynamics simulation analysis

As previously described by Gao et al. [27], topology files for AI-2 and FruA were generated using acpype with the GAFF force field. This process produced itp and gro files that were subsequently used for simulation. To create a neutral aqueous solution system, protein and solvent components were constructed using the amber99sb.ff/tip3p force field. The entire complex was positioned within a cubic periodic box, guaranteeing a minimum distance of 1 nm between the complex and the box edges. Adequate chloride ions were introduced to neutralize the charge of the protein. Energy minimization, NVT temperature control simulations, NPT pressure control simulations, and a 50 ns kinetics simulation were subsequently carried out. The simulations and analyses were performed using the GROMACS 2021.2 software package.

### Animal experiments

#### Determination of the median lethal dose (LD_50_)

All procedures involving animals adhered to the guidelines stipulated in the Administration of Laboratory Animals of China (2017 revision) and received approval from the Experimental Animal Monitoring Committee of Henan University of Science and Technology under the auspices of approval number SKKUIACUC-20–04-14–3. *S. suis* cultured for 8 h was subsequently diluted to concentrations of 10^4^, 10^5^, 10^6^, 10^7^ and 10^8^ CFU/mL. In each group, twenty specific pathogen-free (SPF) female BALB/c mice, aged 4–6 weeks, were intraperitoneally injected. Each group was further subdivided into two segments. At 0 h, 12 h, 24 h, and 48 h, one segment received a 50 μL injection of AI-2 via the tail vein, while the other segment was intravenously injected with 50 μL of PBS. The mortality of the mice was documented after a 7-day period (Figure 6A (panel a)).

#### Tissue bacterial load detection

*The* WT and Δ*luxS* strains were suspended in PBS and further diluted to a concentration of 5 × 10^6^ CFU/mL. SPF female BALB/c mice aged 4–6 weeks were intraperitoneally injected with 200 μL of the bacterial solution in each group. Each group was subsequently subdivided into two segments. At 0 h, 12 h, 24 h, and 48 h, one segment received a 50 μL injection of AI-2 via the tail vein, while the other segment was intravenously injected with 50 μL of PBS as a negative control. All mice were euthanized 3 days later by intravenous injection of pentobarbital. Homogenates of the brain, lung, liver, spleen, and kidney, standardized by weight, were diluted and plated on TSB agar. The CFUs were counted after 24 h (Figure 6A (panel b)).

#### Histological examination

The remaining organs were immobilized in 4% paraformaldehyde (pH = 7) for 36 h. After fixation, the tissue specimens were embedded in paraffin, followed by sectioning into 4 μm-thick slices. Subsequently, these sections were stained utilizing the haematoxylin‒eosin method.

### Statistical analysis

All experiments were repeated three times, and a total of three independent experiments were performed. Student’s *t* test and two-way analysis of variance were performed utilizing GraphPad Prism Software (ver. 9.0, Graph-Pad Software Inc., La Jolla, CA, USA). A significance threshold of *P ≤ 0.05 was used.

## Results

### AI-2 complements *ΔluxS* growth phenotypes

**Figure 1:**
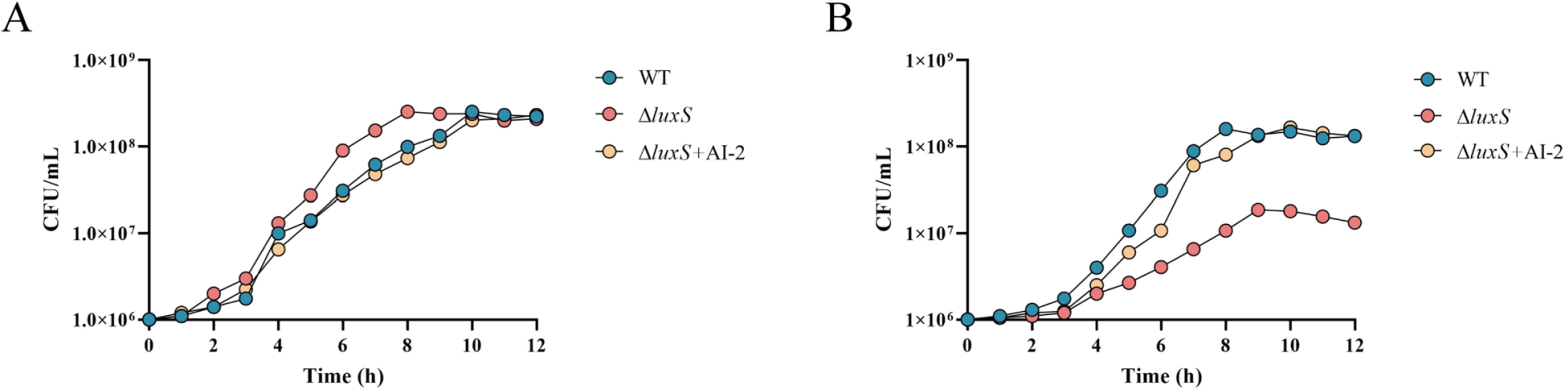
***S. suis*** **growing in CDM Glc (A) and CDM Gal (B).**

*S. suis* primarily colonizes the human upper respiratory tract, which predominantly utilizes galactose as a carbon source [40]. Consequently, we first examined the growth characteristics of the WT and Δ*luxS* strains in CDM supplemented with either glucose (CDM-Glc) or galactose (CDM-Gal). For CDM-Glc, the growth kinetics of both the WT and Δ*luxS* strains were similar, with only minor growth retardation in the WT strain observed (Figure 1A). In contrast, in CDM-Gal, the Δ*luxS* strain exhibited a significantly increased generation time and notably lower final cell density than the WT strain (Figure 1B). This suggests that *luxS* deletion negatively impacts *S. suis* survival in CDM-Gal. To corroborate the role of AI-2 deficiency in this effect, we assessed the ability of AI-2 to mitigate growth defects in Δ*luxS*. Supplementing AI-2 effectively reversed the growth defect in the Δ*luxS* strain in CDM-Gal (Figure 1B). In summary, these findings indicate that extracellular AI-2 can stimulate the metabolism of galactose by *S. suis*, possibly by enhancing bacterial transport and the intracellular processing of galactose.

### AI-2 promotes CPS production

**Figure 2:**
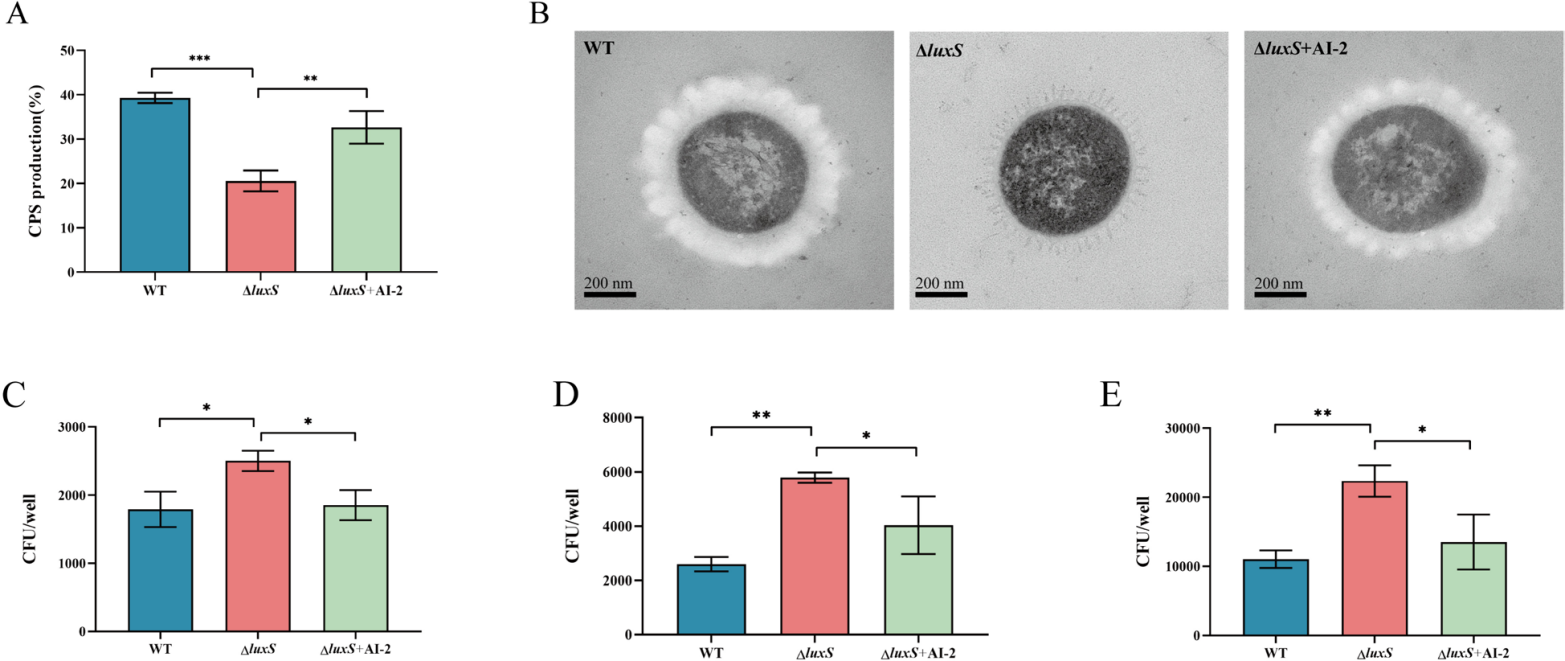
**AI-2 QS affects the production of CPS in *S. suis* grown in the presence of CDM-Glc**. **A** Total CPS production by the WT, Δ*luxS*, and Δ*luxS* + AI-2 strains grown in CDM-Gal. **B** Transmission electron micrographs of bacteria; the bars represent 200 nm. **C**–**E** Number of bacteria engulfed by RAW264.7 cells after 2 h (**C**), 4 h (**D**) and 8 h (**E**) of infection with *S. suis*.

The capacity of *S. suis* to resist macrophage phagocytosis is crucial for its pathogenicity. Consequently, we quantified the CPS content of *S. suis* cultured in CDM-Gal. Compared with that in the WT strain, CPS production in the Δ*luxS* strain decreased by approximately 20%, whereas exogenous AI-2 supplementation increased the CPS content in the Δ*luxS* strain by 12% (Figure 2A). TEM revealed that the WT strain exhibited a tightly enveloped CPS layer, in contrast to the significantly thinner CPS layer in the Δ*luxS* strain (Figure 2B). CPS, known for its antiphagocytic properties, is essential for the infectivity of *S. suis* [41]. Subsequently, we evaluated the bacterial load within macrophages at 2 h, 4 h, and 8 h post infection with *S. suis* to determine the influence of AI-2 on bacterial resistance to phagocytosis. The findings showed that the phagocytosis of the Δ*luxS* strain was significantly greater than that of the WT strain at all time points, suggesting a diminished antiphagocytic capability in the Δ*luxS* strain (Figures 2C, D). Concurrently, supplementation with exogenous AI-2 reduced macrophage phagocytosis by the Δ*luxS* strain, thereby restoring its resistance to macrophage engulfment. This observation aligns with the alterations in CPS levels. These experiments demonstrated that AI-2-regulated growth and CPS-associated activities in *S. suis* are linked to galactose utilization.

### AI-2 QS upregulates galactose metabolism in *S. suis*

Metabolomics has been extensively applied to elucidate the behavioral characteristics of pathogenic microorganisms [42]. This study further explored the regulation of *S. suis* metabolic activity by AI-2 QS through untargeted metabolomics. The PCA score plot demonstrated excellent aggregation and reproducibility within each group (Additional files 2A and B). Notably, there was a distinct separation between the WT and Δ*luxS* strains, underscoring metabolic differences. Similar results were obtained via PLS-DA and OPLS-DA (Additional files 2C and D). These findings confirmed robust reproducibility between replicates and a well-validated model without overfitting (Additional files 2G and H).

**Figure 3:**
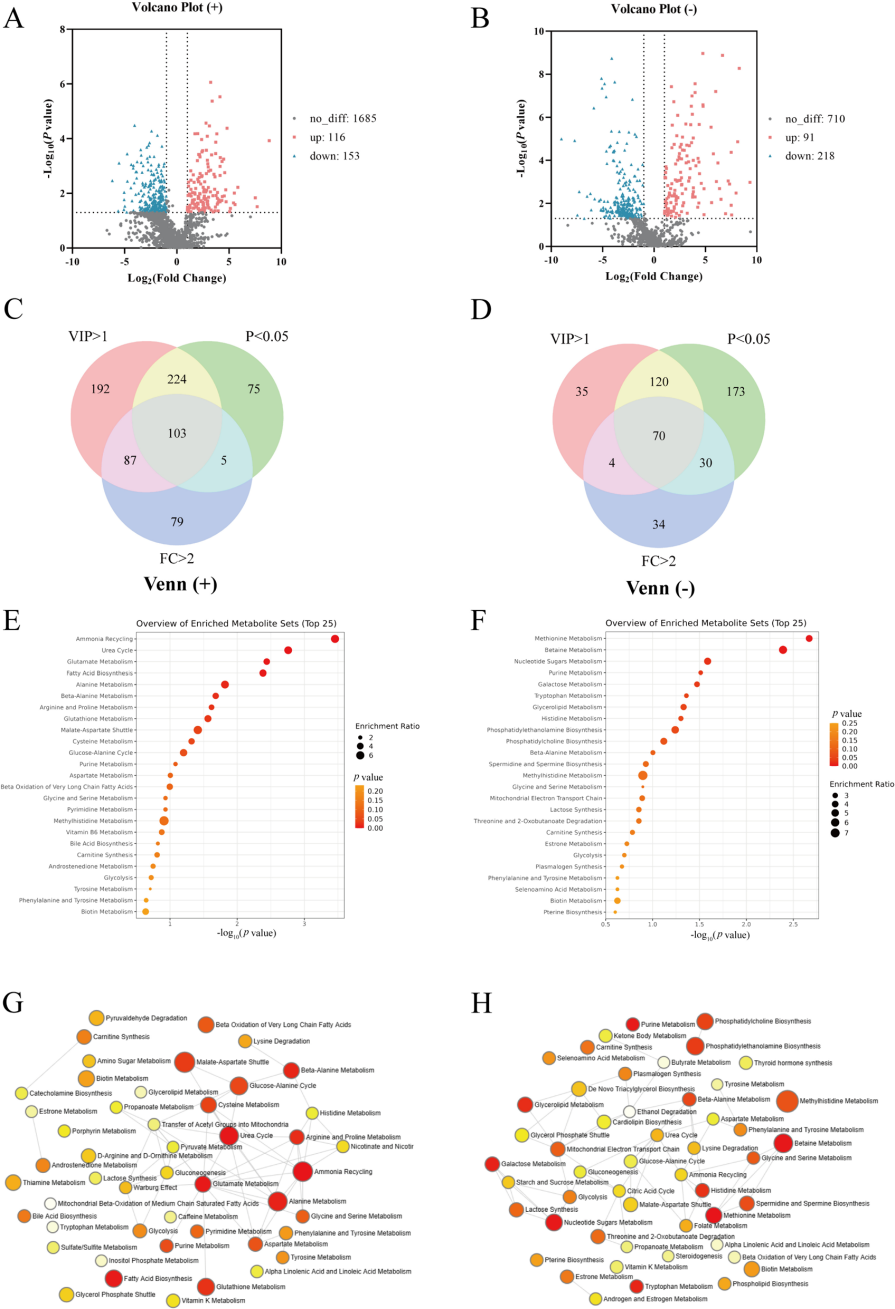
**Metabolomic analysis (Δ*luxS*/WT)**. **A**–**B** Volcano plot of differentially detected metabolites between the WT and Δ*luxS* strains in the ESI ( +) (left) and ESI (−) (right) modes. **C**–**D** Venn diagram of differentially detected metabolites between the WT and Δ*luxS* strains in the ESI ( +) (left) and ESI (−) (right) modes. **E** Detailed upregulated pathways enriched with the differentially detected metabolites. **F** Detailed downregulated pathways enriched in differentially expressed metabolites. **G** Overview of upregulated pathway enrichment of differentially expressed metabolites. **H** Overview of downregulated pathway enrichment of differentially expressed metabolites.

**Figure 4:**
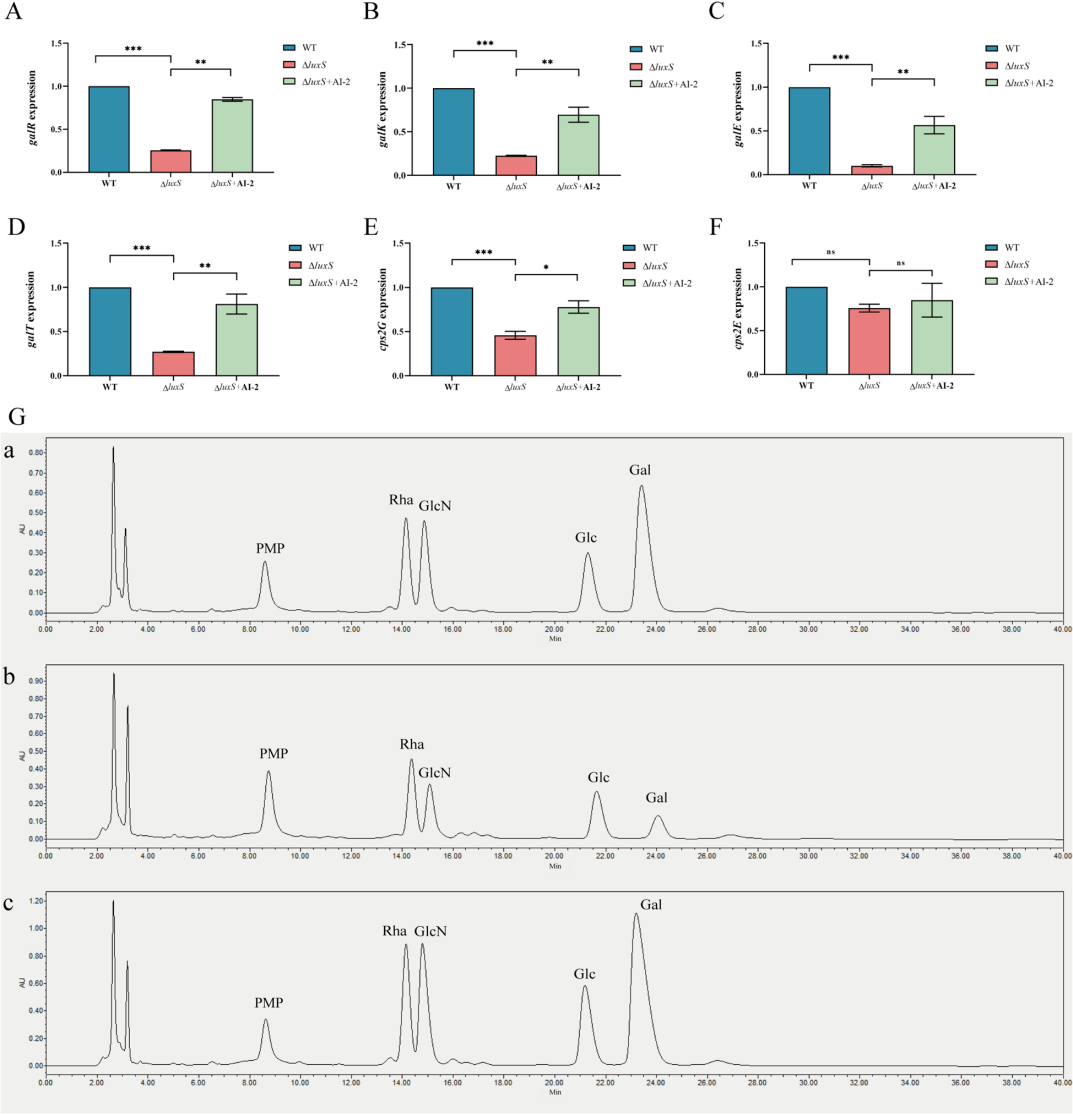
**AI-2 QS regulates galactose metabolism**. **A**–**F** Relationships between the expression of *galR*, *galE*, *galK*, *galT*, *cps2G*, and *cps2E* in *S. suis* grown in CDM-Gal with or without 4 μM AI-2 were quantified by qRT‒PCR with 16S rRNA as an internal standard. *P < 0.05; **P < 0.01; ***P < 0.001 (Student’s unpaired *t* test). **G**, (panel a) The CPS components of *S. suis* (WT). **G**, (panel b) The CPS components of *S. suis* (Δ*luxS*). **G**, (panel c) The CPS of *S. suis* (Δ*luxS* + AI-2).

Using a fold change (FC) threshold greater than 2, we identified 116 upregulated and 153 downregulated compounds in positive ion mode (Figure 3A). In negative ion mode, 91 upregulated and 218 downregulated compounds were identified (Figure 4B). Further refinement using criteria such as VIP values > 1, *p* < 0.001, and FC > 2 revealed 103 and 70 distinct metabolites in positive and negative ion modes, respectively (Figures 3C, D). Significantly upregulated and downregulated metabolites were separately extracted for enrichment analysis. The analysis indicated that compared with those in the WT strain, the upregulated metabolites in the Δ*luxS* strain were primarily involved in glutamate metabolism, fatty acid biosynthesis, and alanine metabolism. Conversely, downregulated metabolites were enriched in pathways such as methionine metabolism, betaine metabolism, nucleotide sugar metabolism, purine metabolism, and galactose metabolism (Figures 3E, F). We further analysed the expression of genes in the Leloir pathway, namely, *galR* (transcriptional regulator), *galK* (galactokinase), *galE* and *galT* (galactose-1-phosphate uridyltransferase), which are central to galactose metabolism. The results demonstrated significantly lower expression of *galR*, *galE*, *galK*, and *galT* in the Δ*luxS* strain than in the WT strain (Figures 4A–D), suggesting that AI-2 QS impacts galactose transport and breakdown. Additionally, supplementation with AI-2 increased the expression of Leloir pathway genes, further confirming that the deficiency in galactose metabolism due to *luxS* deletion is mediated by AI-2 QS via the modulation of the Leloir pathway.

Galactose serves as the preferred carbon source for pulmonary respiratory pathogens and is a critical raw material for bacterial capsule synthesis. Bacteria generate glucose-1-phosphate (G1P) via the Leloir pathway, subsequently producing various UDP-activated sugar precursors essential for CPS synthesis. This process facilitates bacterial capsule formation and enhances resistance against the immune system [7, 43]. Consequently, we also examined the expression of CPS side-chain transfer regulatory genes (*cps2G* and *cps2E*) linked to the Leloir pathway. Our findings indicate that AI-2 QS significantly regulates *cps2G*, but not *cps2E*, in *S. suis* (Figures 2D, E). The *cps2G* gene is responsible for transferring galactose to the side chain of *S. suis* CPS [44, 45]. Consequently, we employed HPLC to determine the molar ratios of the individual sugar components in CPS. Deletion of *luxS* led to reduced molar proportions of Gal and GlcN (Table 2) (Figure 4G). Notably, the most significant decrease was observed for galactose, suggesting that Leloir pathway inhibition results in reduced synthesis of galactose side chain precursors. Overall, AI-2 QS enhances CPS synthesis by upregulating galactose metabolism, thereby increasing the transfer of side chain monosaccharides, which in turn augments the virulence of *S. suis*.

**Table 2:** **Molar ratio of each monosaccharide of** ***S. suis*** **CPS.**

	Rha	Glc	Gal	GlcN
WT	1	1.1	2.5	0.9
Δ*luxS*	1	1.1	1.9	0.7
Δ*luxS* + AI-2	1	1.1	2.4	0.9

### Molecular docking and dynamic simulation

**Figure 5:**
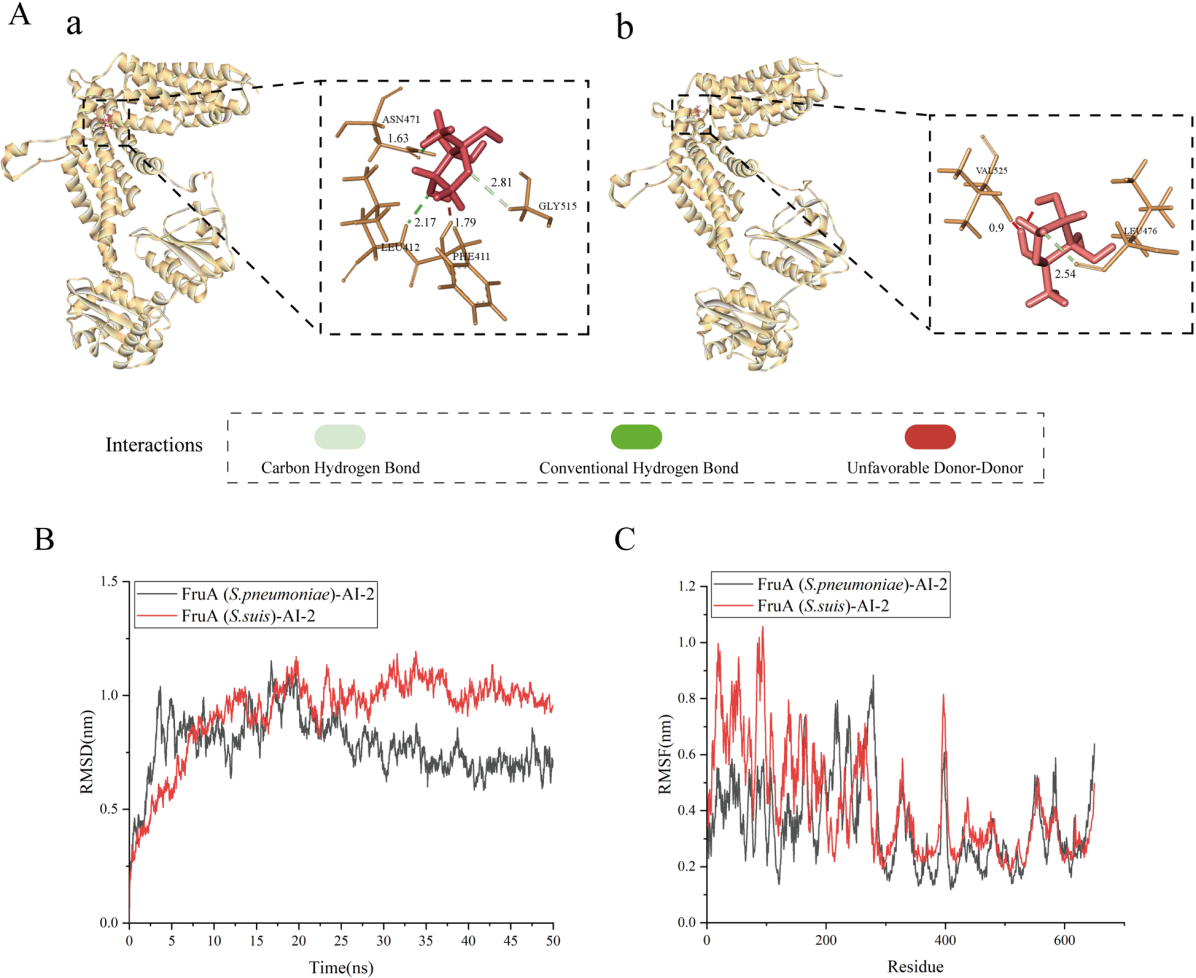
**Docking analysis**. **A**, (panel a) Molecular docking results between AI-2 and the FruA protein model of *S. suis*. **A**, (panel b) Molecular docking results between AI-2 and the FruA protein model of *S. pneumoniae*. **B** RMSD distribution map. **C** RMSF distribution map. The black line represents the complex of FruA (*S. pneumoniae*) and AI-2, while the red line represents the complex of FruA (*S. suis*) and AI-2.

AI-2, as a signalling molecule, either activates or inhibits bacteria-related activities by interacting with specific target proteins. The fructose PTS component IIABC, known as FruA, plays a crucial role in AI-2 signalling in *S. pneumoniae* [44]. FruA is responsible for recognizing and internalizing AI-2, potentially leading to its phosphorylation to form AI-2-P. Subsequently, AI-2-P influences the galactose transcriptional regulatory protein GalR, modulating its activation or repression within the Gal operon. This cascade mechanism is pivotal in regulating galactose metabolism in *S. pneumoniae*. SWISS-MODEL was used to construct a FruA (fructose PTS transporter) 3D model. The model with high sequence similarity is considered to be the best [36]. Therefore, FruA (V6Z463.1. A) (Additional file 3) and FruA (Q8DQ95.1. A) (Additional file 4) were selected for further analysis. Most of the amino acid scores of the model were greater than 0.6 in the local mass map (Additional files 5-6A). In the comparison score map (Additional files 5-6B and C), the QMEANZ score reflects the quality of the model, and the closer it is to 0, the better the match between the tested protein and the template protein. The red star represents the model built. Furthermore, the Ramachandran plots show that 95.83% (Additional file 5F) and 95.99% (Additional file 6F) of the residues exist in favourable regions, indicating that the protein structure is highly reasonable. In summary, the FruA models are of good quality. Subsequently, we compared the binding affinity of these proteins with that of the AI-2 signalling molecule. The docking results indicated that AI-2 binds to FruA of *S. suis*, showing a greater affinity than to FruA of *S. pneumoniae* (Additional file 7). The interaction between AI-2 and FruA in *S. suis* involves conventional hydrogen bonds and carbon‒hydrogen bonds. As depicted in Figure 5, AI-2 forms conventional hydrogen bonds with ASN471 and LEU412 in FruA of *S. suis* (Figure 5A, panel a). The proximity of hydrogen donors and acceptors within a distance of less than 3 Å indicates the likelihood of strong hydrogen bond formation [46, 47]. Conversely, AI-2 forms only one carbon‒hydrogen bond with FruA in *S. pneumoniae* (Figure 5A, panel b). This indicates that compared to the FruA protein of *S. pneumoniae*, AI-2 has a better docking effect with the FruA protein of *S. suis*.

We simulated the binding dynamics of FruA and AI-2 over 50 ns using the root mean square deviation (RMSD) and root mean square fluctuation (RMSF). In MD simulations, RMSD measures the equilibrium and flexibility of protein–small molecule interactions and monitors the distances between protein backbones and atoms. Minor fluctuations and lower RMSD values suggest a more stable interaction between the protein and its ligand [48]. The results indicated that the final RMSD values for AI-2 binding to FruA were approximately 0.7 nm and 1.0 nm for *S. pneumoniae* and *S. suis*, respectively (Figure 5B). However, the lower final RMSD of the FruA (*S. pneumoniae*)-AI-2 complex compared to that of the FruA (*S. suis*)-AI-2 complex does not necessarily imply a stronger binding affinity. The stability of the FruA (*S. suis*)-AI-2 complex is attributed to the lower fluctuation observed (0.25 nm) during the simulation compared to that of the FruA (*S. pneumoniae*)-AI-2 complex, which exhibited a larger fluctuation of 0.5 nm (Figure 5B). RMSF predicts atomic displacements under specific conditions, indicating changes in protein flexibility upon ligand binding and determining residue fluctuations during MD simulations. The data presented in these figures demonstrate greater flexibility in protein fragments within the 0–200 region of the FruA (*S. suis*)-AI-2 complex than within the FruA (*S. pneumoniae*)-AI-2 complex, suggesting significant amino acid activity during the binding process of *S. suis* to AI-2 (Figure 5C). In the remaining sections, the effects of FruA (*S. suis*)-AI-2 and FruA (*S. pneumoniae*)-AI-2 were consistent.

### Exogenous AI-2 increases *S. suis* virulence

**Figure 6:**
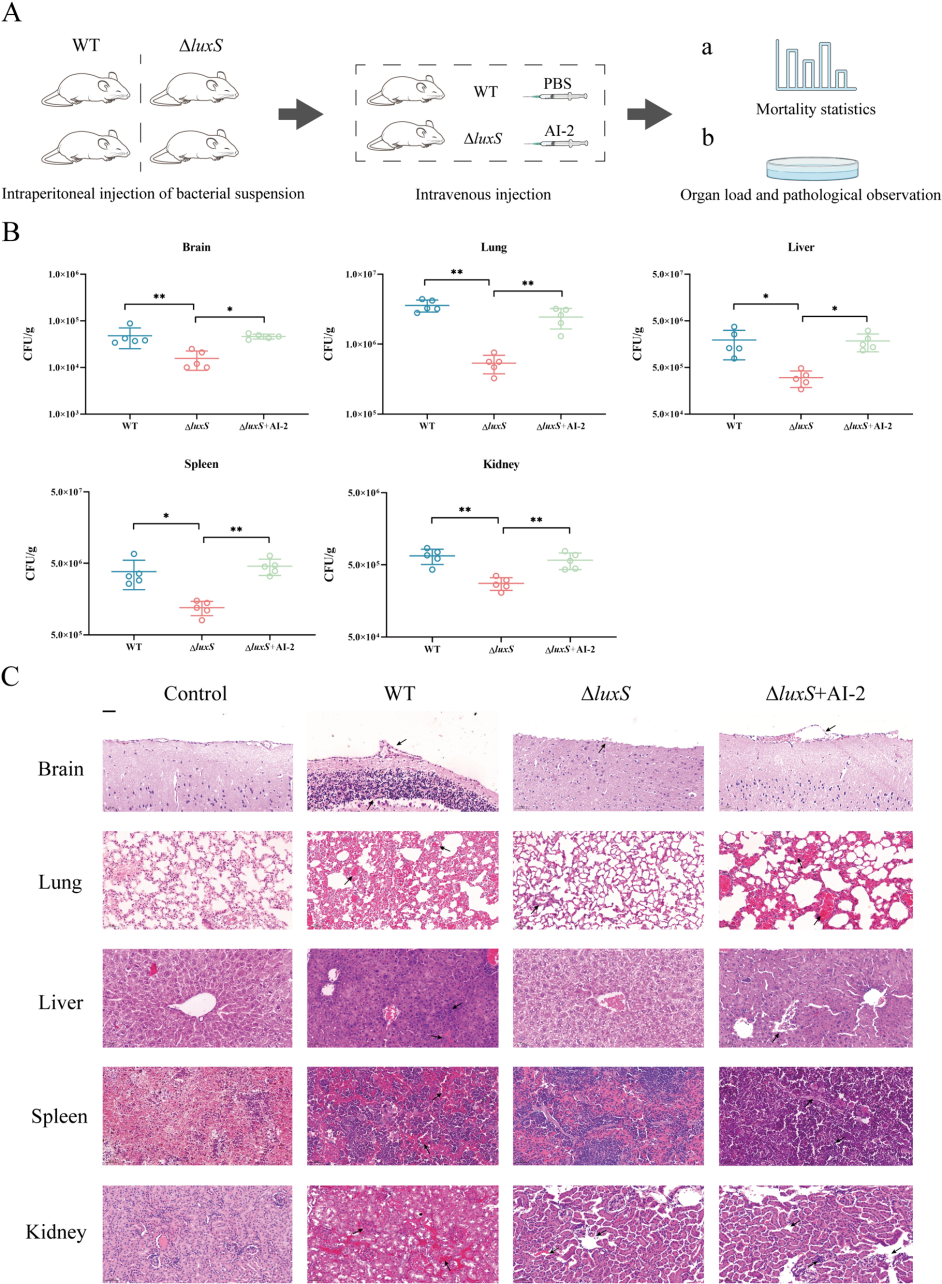
**AI-2-regulated galactose metabolism-dependent CPS synthesis affects the infectivity of *S. suis****.* **A** Steps of mouse infection. (Panel a) Median lethal dose measurement experiment. (panel b) Tissue bacterial load measurement and pathological observation. **B** Bacterial load in various tissues and organs of mice. **C** Pathological changes in the brain, heart, lung, liver, spleen and kidney. Scale bar (black scale): 50 μm.

Our previous research indicated that the Δ*luxS* strain of *S. suis* displayed reduced virulence in zebrafish compared to the WT strain [49]. In this study, LD_50_ experiments revealed that the Δ*luxS* strain also exhibited decreased virulence in mice, a condition reversible by external AI-2 supplementation. The LD_50_ for the WT strain was 5.77 × 10^5^ CFU, in contrast to the 8.91 × 10^6^ CFU for the Δ*luxS* strain, which was 15 times greater than that of the WT strain. Following the addition of exogenous AI-2, the LD_50_ for the Δ*luxS* strain decreased to 5.54 × 10^5^ CFU, comparable to that of the WT strain (Table 3). Organ bacterial load experiments demonstrated that mice infected with the Δ*luxS* strain had significantly lower bacterial loads across various organs than those infected with the WT strain. External AI-2 supplementation resulted in increased bacterial loads in all tested organs (Figure 6B).

**Table 3:** **Calculation of LD**_**50**_** of *S. suis* in mice.**

Group	CFU per mouse				Mortality				LD_50_/CFU
WT	2.979 × 10^7^	2.967 × 10^6^	2.587 × 10^5^	3.173 × 10^4^	10/10	8/10	3/10	1/10	5.77 × 10^5^
△*luxS*	2.832 × 10^8^	2.776 × 10^7^	2.432 × 10^6^	3.203 × 10^5^	9/10	7/10	4/10	0/10	8.91 × 10^6^
△*luxS* + AI-2	2.776 × 10^7^	2.335 × 10^6^	2.967 × 10^5^	2.857 × 10^4^	9/10	8/10	3/10	2/10	5.54 × 10^5^

The pathological findings are presented in Figure 6C. Mice in the WT-infected group displayed meningeal congestion and shedding, accompanied by inflammatory cell infiltration. Brain tissue lesions in the Δ*luxS* strain-infected group were milder and were characterized by limited inflammatory cell infiltration. The lesions in the Δ*luxS* + AI-2 group were similar to those in the WT group. In lung tissues, the WT-infected group exhibited severe alveolar structure disruption, characterized by shedding of epithelial cells, the presence of inflammatory cells, and red blood cell infiltration in alveolar spaces. Mice infected with the Δ*luxS* strain exhibited less severe lung tissue damage, with limited mucus and detached epithelial cells in some alveoli. In the Δ*luxS* + AI-2 group, lung tissue damage was exacerbated, characterized by alveolar spaces filled with red blood cells and neutrophils, disrupting alveolar structures. In the spleen, WT-infected mice experienced severe congestion, significant inflammatory cell infiltration, white pulp atrophy, and reduced lymphocyte counts. Conversely, mice infected with the Δ*luxS* strain exhibited milder splenic tissue damage. However, spleen tissue damage in the Δ*luxS* + AI-2 group mirrored that in the WT group, indicating a more severe condition. In WT-infected mice, liver tissues showed disrupted lobules, central vein and hepatic sinus congestion, extensive inflammatory cell infiltration, and hepatocyte granular or vacuolar degeneration. Mice infected with the Δ*luxS* strain showed liver lobule disruption but relatively intact hepatocytes, central vein congestion, and less pronounced hepatic sinus congestion. The Δ*luxS* + AI-2 group exhibited liver lobule disruption with infiltration of neutrophils, lymphocytes, and macrophages in the hepatic sinus. In kidney tissues, WT-infected mice exhibited fragmented glomerular capillary endothelial cells, central granulocyte infiltration, vascular rupture, and reduced renal tubule volume. Renal tubular epithelial cells exhibited granular degeneration with thread-like reddish cytoplasm and inflammatory cell infiltration. Mice infected with the Δ*luxS* strain displayed swelling of glomerular capillary endothelial cells, vascular congestion, and inflammatory cell infiltration in renal tubules. In the Δ*luxS* + AI-2 group, renal pathology resembled that of the WT-infected group, with wrinkled glomeruli, inflammatory cell infiltration, and granular degeneration in renal tubular epithelial cells.

## Discussion

*S. suis*, a significant zoonotic pathogen, is typically classified based on its serotypes. The distribution of *S. suis* serotypes in clinical cases varies geographically; however, serotype 2 strains predominate in swine and human infections globally. This has led to serotype 2 being historically considered the most prevalent and virulent [50]. QS plays a vital role in bacterial behavioral regulation, with AI-2 QS, which is based on AI-2 signalling molecules, acknowledged universally as a “language” for bacterial communication [13]. However, it remains uncertain whether AI-2 QS solely activates the methyl cycle or participates in other bacterial metabolic activities. Furthermore, limited knowledge of AI-2 receptors in gram-positive bacteria impedes research into the transduction of AI-2 signalling molecules. Nonetheless, it is evident that *luxS* is central to the regulatory system governing bacterial life activities. The absence of *luxS* in *S. suis* impacts several functions, notably antibiotic tolerance and virulence [26, 51, 52]. Presently, the in vitro synthesis of DPD has advanced, allowing for its self-cyclization to form active AI-2, which is useful in phenotypic complementation experiments for the Δ*luxS* strain. Synthetic AI-2 is utilized to correct growth and CPS production deficiencies in *S. suis* and to rectify the impaired galactose metabolism of the Δ*luxS* strain.

This study highlights the significance of AI-2 QS in the utilization of galactose by *S. suis*. In the upper respiratory tract, galactose is scarce, making galactose the principal carbon source for pathogens [53]. For respiratory pathogens such as *S. suis*, the ability to utilize galactose is crucial for survival and successful infection. The growth phenotypes of *S. suis* were compared in media supplemented with galactose as the sole carbon source (CDM-Gal), revealing that *luxS* deletion results in a galactose metabolic defect, which is reversible with exogenous AI-2. This observation aligns with the noted reduction in CPS synthesis in the Δ*luxS* strain. TEM further confirmed this phenomenon, indicating that a CPS defect in Δ*luxS* was rectifiable with exogenous AI-2. In invasive bacterial infections, the interplay between the bacterial capsule and the host immune system is pivotal in determining the infection outcome [54]. CPSs may mask antigen binding sites on the bacterial surface, thereby hindering the attachment of immunoglobulin G (IgG) to bacteria. Feng et al. noted that CPS deficiency increases the susceptibility of *S. suis* to phagocytosis, accelerating the release and secretion of proinflammatory cytokines and interleukin-8 (IL-8) in the host [4]. Moreover, the capsule of *Francisella tularensis* (*F. tularensis*) inhibited the increase in lactic acid secretion triggered by R848. This inhibition disrupts the shift in phagocytic metabolism from oxidative phosphorylation to glycolysis, ultimately reducing cytokine secretion [55]. Cell phagocytosis tests revealed that compared with that in the WT strain, the resistance of the Δ*luxS* strain to CPS deficiency in RAW264.7 cells decreased, leading to increased phagocytosis of Δ*luxS*.

**Figure 7:**
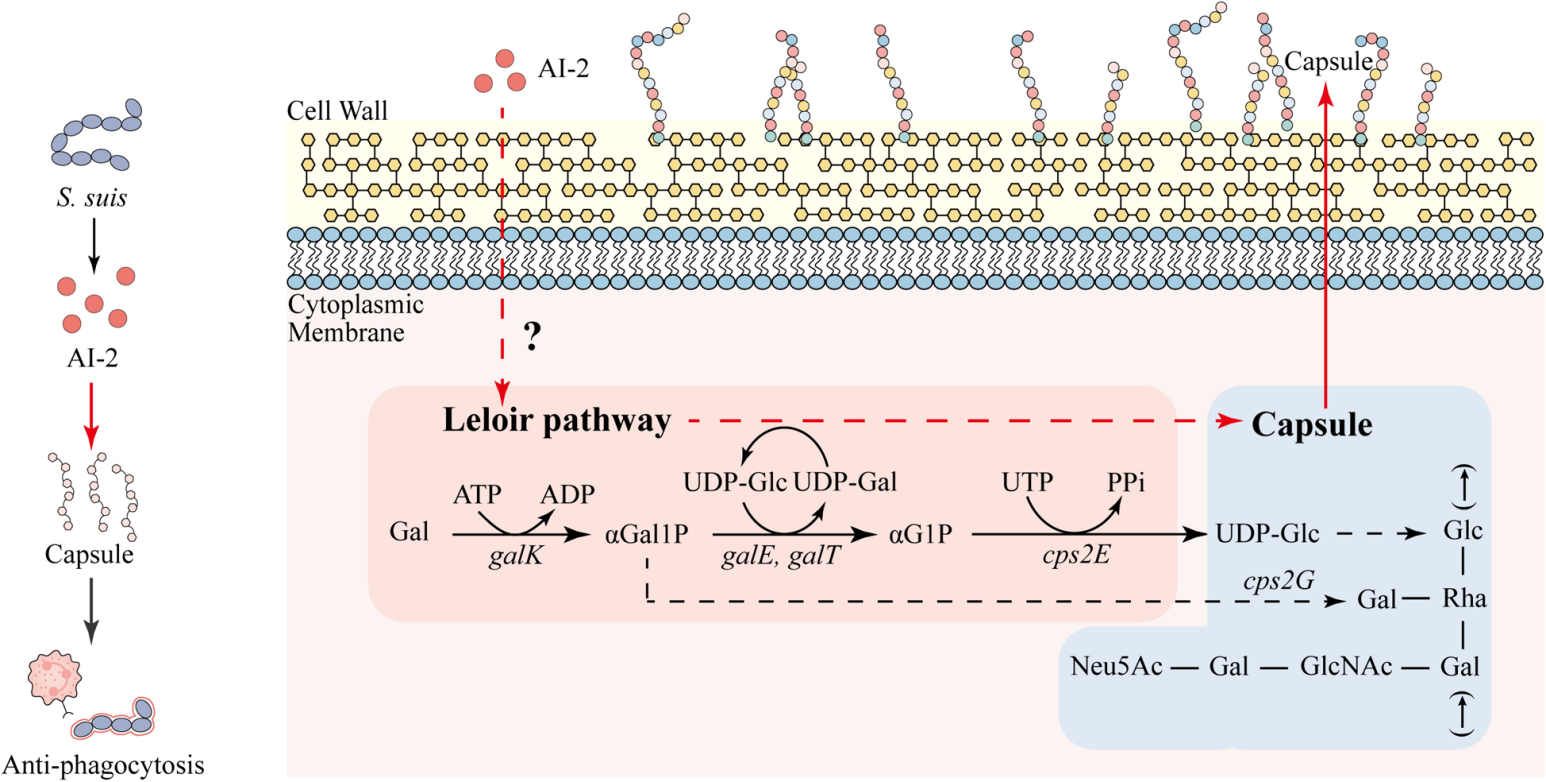
**AI-2 QS regulates galactose metabolism and promotes CPS synthesis.**

These observations indicate a strong correlation between AI-2 QS and galactose metabolism in *S. suis*. The metabolic differences between the WT and Δ*luxS* strains were analysed using HPLC–MS/MS to study their growth in CDM-Gal. Enrichment analysis revealed downregulation of the galactose metabolic pathway in Δ*luxS* compared to that in WT. Further analysis of the impact of AI-2 QS on Leloir pathway-related genes revealed significant downregulation of *galR*, *galE*, *galK*, and *galT* in Δ*luxS* compared to that in WT. Gal is converted to G1P via the Leloir pathway, yielding the UDP-activated sugar precursors essential for CPS synthesis [25, 40]. Furthermore, variations in the configuration of the CPS, including the arrangement and proportion of monosaccharides, influence its chemical structure and functional activity. For example, in *Vibrio cholerae*, the substitution of the α-D-Glc moiety in the polysaccharide component with α-D-GlcNAc increases viscosity, promoting biofilm formation [56]. Consequently, the measurement of CPS components revealed a reduced ratio of Gal and GlcN in CPS. Investigation of the expression of CPS side chain regulatory genes linked to galactose metabolism revealed a notable decrease in the expression of *cps2G*, which is responsible for the transfer of Gal to the side chain, compared to that of *cps2E*, which is responsible for the synthesis of the main chain of CPS. This finding supports our hypothesis that *S. suis* can influence CPS synthesis by modulating galactose metabolism and the expression of CPS side chain transfer genes via AI-2 QS (Figure 7).

The mechanism of AI-2 signal transmission within cells is crucial, especially since AI-2 has been shown to enhance CPS production and virulence in *S. suis*. The AI-2 receptors identified to date include LuxP, LsrB, and TlpB in gram-negative bacteria and RbsB in gram-positive bacteria [57–59]. LuxP in *Vibrio harveyi* and LsrB in *Escherichia coli* have been extensively studied. In *Vibrio harveyi*, LuxP, a periplasmic receptor, binds AI-2 to form an AI-2/LuxP complex, inhibiting LuxQ autophosphorylation. This inhibition decreases *Qrr1-5* expression and mediates *luxR* mRNA translation, affecting bioluminescence [60]. In *Salmonella Typhimurium*, LsrB binds AI-2, and the resulting AI-2/LsrB complex is internalized through an ABC transport system. This complex is phosphorylated by the kinase LsrK, increasing the inhibitory effect of LsrR on the lsr operon and leading to the expression of *lsrK*, *lsrR*, and *lsrACDBFGE* [20]. Nevertheless, a substantial gap remains in our understanding of the recognition and transmission of LuxS-mediated AI-2 in *S. suis*. Drawing on the study by Trappetti et al. in *S. pneumoniae*, we speculated that AI-2 recognition in *S. suis* might involve FruA [40]. Consequently, we compared the affinity of AI-2 for FruA in both *S. suis* and *S. pneumoniae*. The results indicated that AI-2 exhibited a favourable docking score with *S. suis* FruA compared to *S. pneumoniae*. Moreover, molecular dynamics simulations revealed that the binding of AI-2 and FruA in *S. suis* was more stable than that in *S. pneumoniae*. The stable fluctuation of FruA binding in *S. suis* indicates that FruA may be the membrane surface receptor of AI-2 in this species. However, more experiments are needed to clarify the AI-2 receptor and its mode of action in *S. suis*.

Our findings provide strong evidence that *S. suis* enhances galactose utilization via AI-2 QS, especially in the biosynthesis of CPS precursors. This increased CPS production leads to enhanced macrophage resistance and greater tissue invasion in *S. suis*. Furthermore, our study provides initial support for the hypothesis that the FruA protein in *S. suis* acts as a receptor for AI-2 signalling molecules. Considering the high conservation of FruA in gram-positive bacteria, FruA is a potential target for novel antibacterial agent development. Receptor proteins such as FruA could be exploited to design or identify new antibacterial drugs that weaken bacterial colonization and prevent systemic infection. These agents are anticipated to lack bactericidal effects, ensuring a controlled impact on the host’s microbial ecology. Moreover, these agents are not expected to exert selective pressure, leading to the evolution of resistance.

### Supplementary Information

**Additional file 1. Standard curve equation.**

**Additional file  2. Multivariate statistical analysis of metabolomic profile. PCA score plot (A-B). PLS-DA analysis (C-D). OPLS-DA (E-F).** There are six replicates in each group. Red and green represent WT and Δ*luxS* groups, respectively. Permutation test (G-H).

**Additional file 3. Structures of FruA protein models of** ***S. suis.***

**Additional file 4. Structures of FruA protein models of** ***S. pneumoniae.***

**Additional file 5. Quality evaluation results of FruA model of** ***S. suis*** **(A-D). (A) Local quality estimate. (B) QMEAN4 scores of the comparison with a nonredundant set of PDB structures. (C) QMEAN Z Scores. (D) Ramachandran plots.**

**Additional file 6. Quality evaluation results of FruA model of** ***S. pneumoniae*** **(A-D). (A) Local quality estimate. (B) QMEAN4 scores of the comparison with a nonredundant set of PDB structures. (C) QMEAN Z Scores. (D) Ramachandran plots.**

**Additional file 7. Docking results of AI-2 with FruA.**

## Data Availability

The datasets generated during and analysed during the study are available from the corresponding author upon reasonable request.
