# Supplementary figures and images for: AI-2 quorum sensing-induced galactose metabolism activation in Streptococcus suis enhances capsular polysaccharide-associated virulence
Source: Vet Res. 2024 Jun 17;55:80. doi: 10.1186/s13567-024-01335-5 (PMC11184709; doi:10.1186/s13567-024-01335-5)

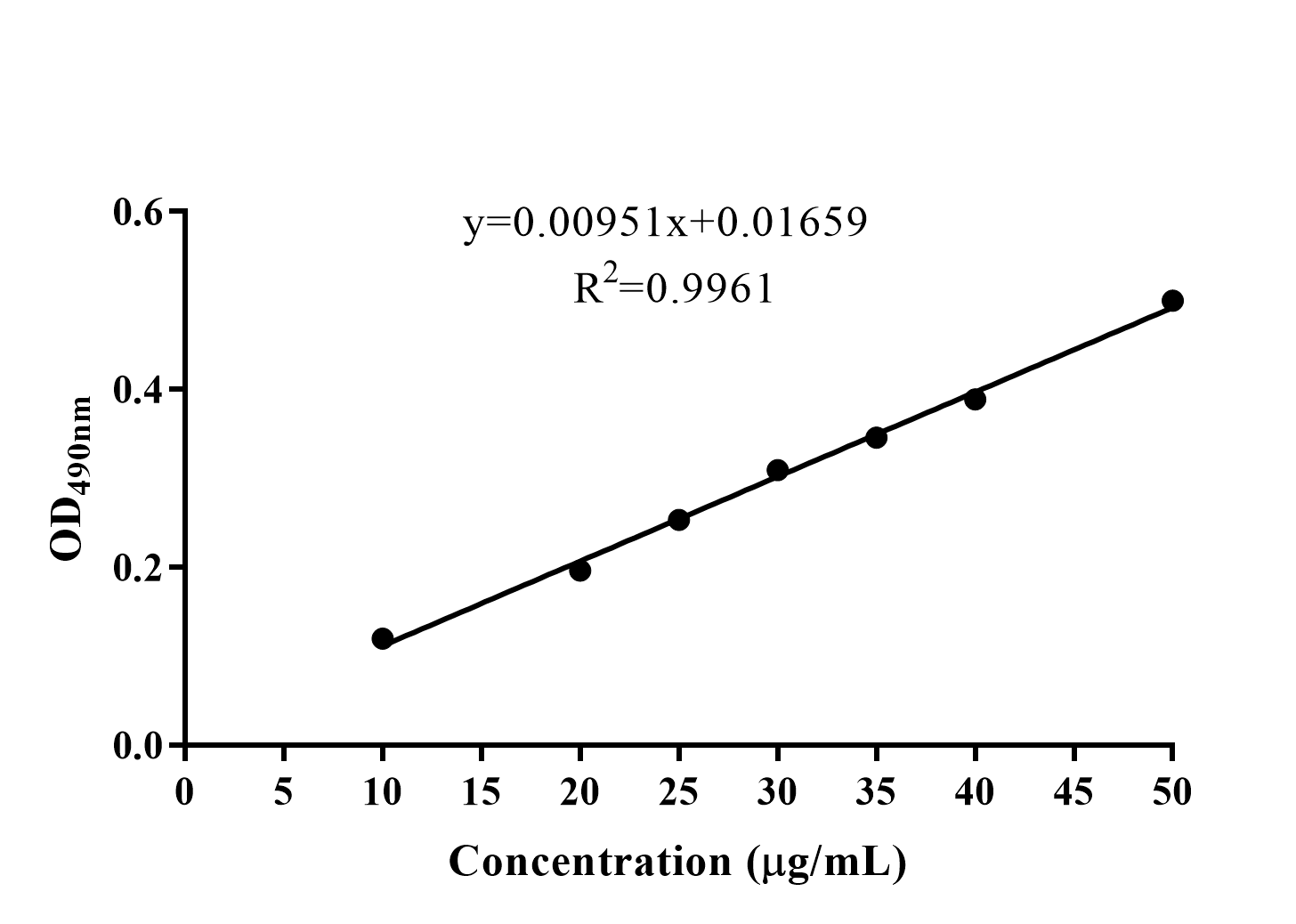

Supplement: Supplementary file 1 — Additional file 1. Standard curve equation. [file 13567_2024_1335_MOESM1_ESM.tif]

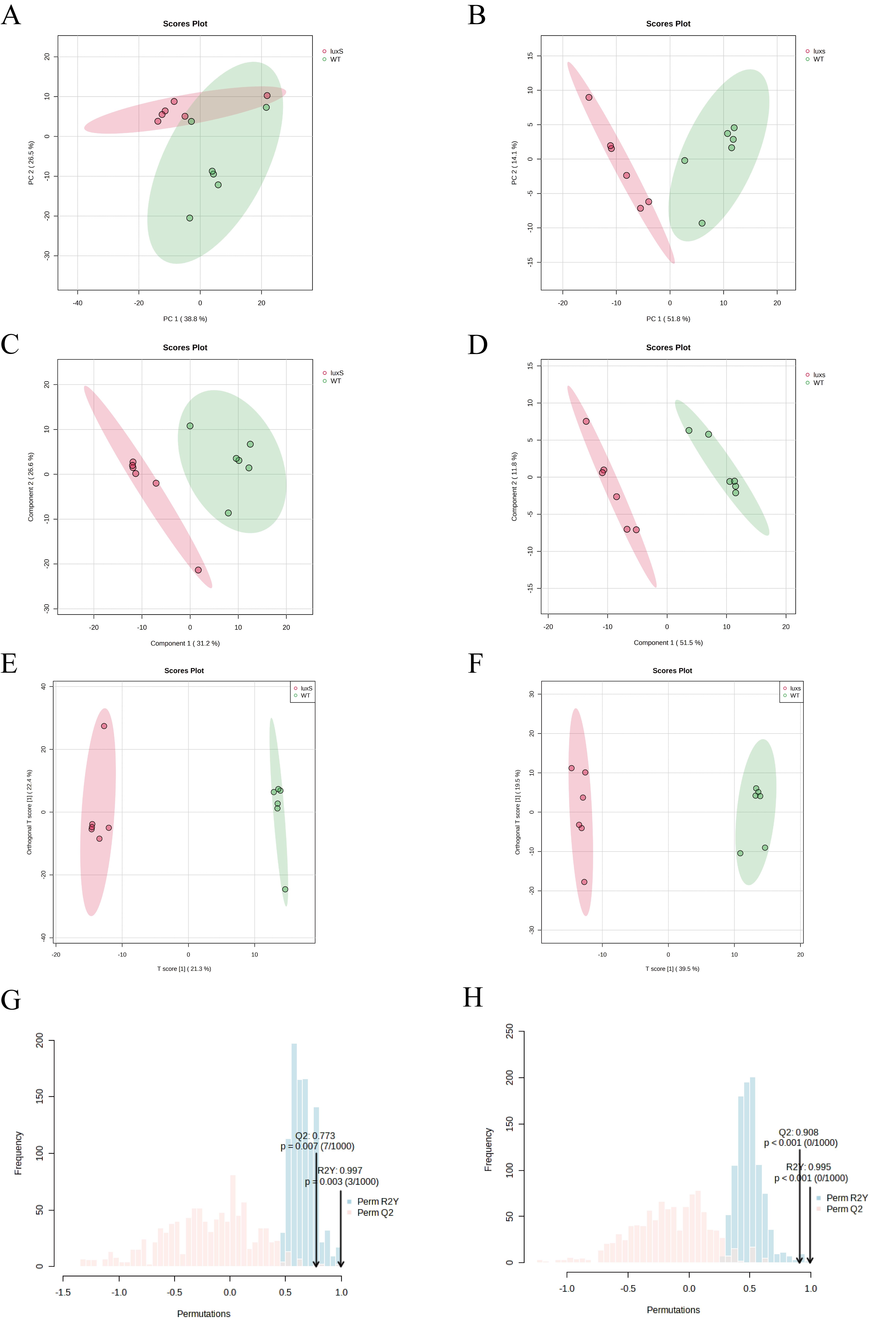

Supplement: Supplementary file 2 — Additional file 2. Multivariate statistical analysis of metabolomic profile. PCA score plot (A-B). PLS-DA analysis (C-D). OPLS-DA (E-F). There are six replicates in each group. Red and green represent WT and ΔluxS groups, respectively. Permutation test (G-H). [file 13567_2024_1335_MOESM2_ESM.tif]

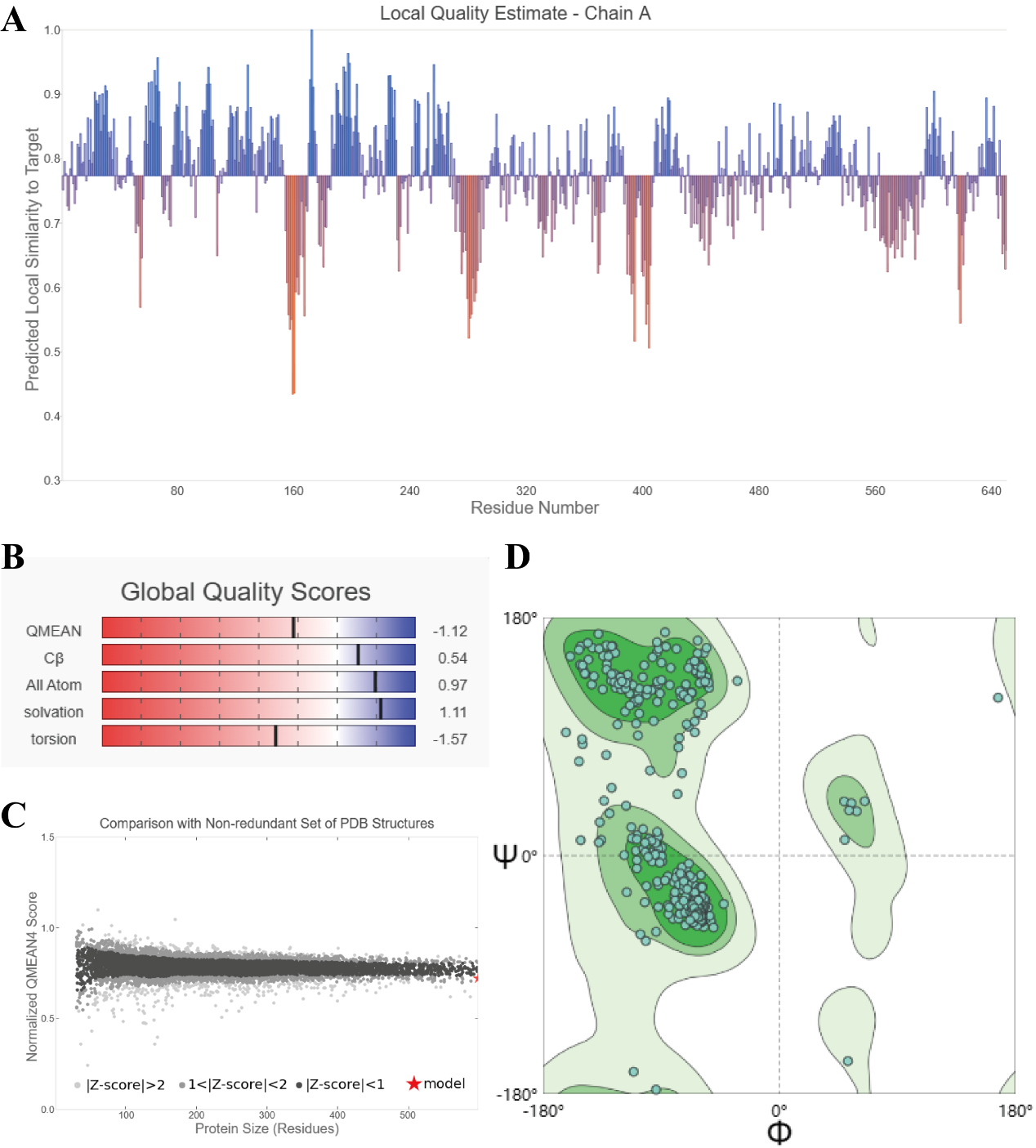

Supplement: Supplementary file 5 — Additional file 5. Quality evaluation results of FruA model of S. suis (A-D). (A) Local quality estimate. (B) QMEAN4 scores of the comparison with a nonredundant set of PDB structures. (C) QMEAN Z Scores. (D) Ramachandran plots. [file 13567_2024_1335_MOESM5_ESM.tif]

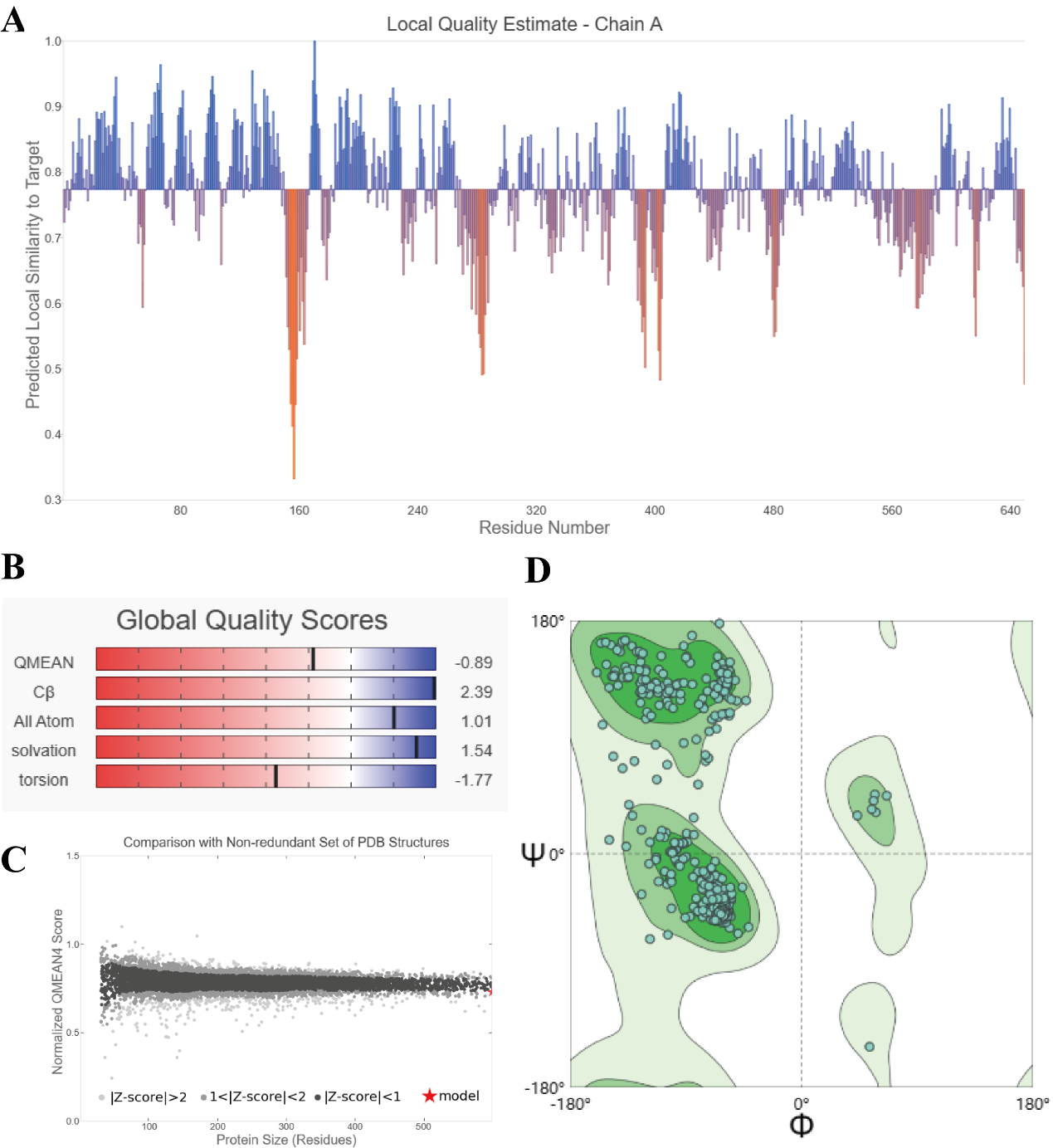

Supplement: Supplementary file 6 — Additional file 6. Quality evaluation results of FruA model of S. pneumoniae (A-D). (A) Local quality estimate. (B) QMEAN4 scores of the comparison with a nonredundant set of PDB structures. (C) QMEAN Z Scores. (D) Ramachandran plots. [file 13567_2024_1335_MOESM6_ESM.tif]
