# Supplementary material for: AI-2 quorum sensing-induced galactose metabolism activation in Streptococcus suis enhances capsular polysaccharide-associated virulence
Source: Vet Res. 2024 Jun 17;55:80. doi: 10.1186/s13567-024-01335-5 (PMC11184709; doi:10.1186/s13567-024-01335-5)
Supplement: Supplementary file 3 — Additional file 3. Structures of FruA protein models of S. suis. [file 13567_2024_1335_MOESM3_ESM.docx]

**Additional file 3 Structures of FruA protein models of *S. suis.***

| Protein type | Template | Description | Sequence similarity (%) | Sequence identity (%) | GMQE Score | QMEAN score | Model |
| --- | --- | --- | --- | --- | --- | --- | --- |
| FruA | V6Z463.1.A | PTS fructose transporter subunit IIC | 53 | 79.57 | 0.87 | -1.12 | 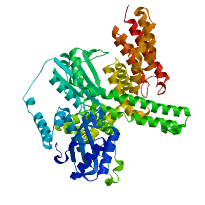 |
|  | 3qnq.1.A | PTS system, cellobiose-specific IIC component | 27 | 16.19 | 0.23 | -9.75 | 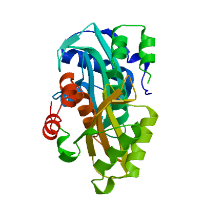 |
|  | 5iws.1.A | PTS system glucose-specific IIBC component | 28 | 16.94 | 0.24 | -8.49 | 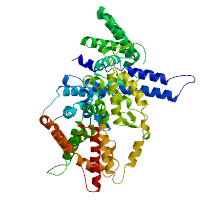 |
|  | 3urr.1.A | PTS IIA-like nitrogen-regulatory protein PtsN | 33 | 22.15 | 0.13 | -2.45 | 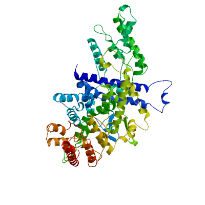 |
