# Supplementary material for: AI-2 quorum sensing-induced galactose metabolism activation in Streptococcus suis enhances capsular polysaccharide-associated virulence
Source: Vet Res. 2024 Jun 17;55:80. doi: 10.1186/s13567-024-01335-5 (PMC11184709; doi:10.1186/s13567-024-01335-5)
Supplement: Supplementary file 4 — Additional file 4. Structures of FruA protein models of S. pneumoniae. [file 13567_2024_1335_MOESM4_ESM.docx]

**Additional file 4. Structures of FruA protein models of *S. pneumoniae.***

| Protein type | Template | Description | Sequence similarity (%) | Sequence identity (%) | GMQE Score | QMEAN score | Model |
| --- | --- | --- | --- | --- | --- | --- | --- |
| FruA | Q8DQ95.1.A | Fructose specific-phosphotransferase system IIBC component | 60 | 99.85 | 0.88 | -0.89 | 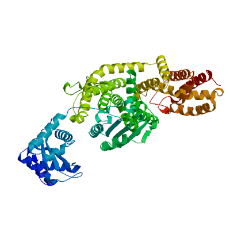 |
|  | 5iws.1.A | PTS system glucose-specific IIBC component | 27 | 14.83 | 0.26 | -8.26 | 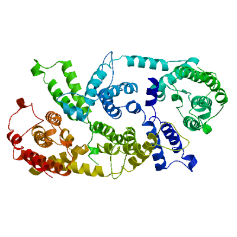 |
|  | 3qnq.1.A | PTS system, cellobiose-specific IIC component | 27 | 15.67 | 0.21 | -8.88 | 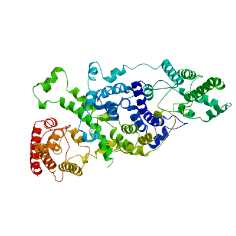 |
|  | 3urr.1.A | PTS IIA-like nitrogen-regulatory protein PtsN | 34 | 26.17 | 0.14 | -2.6 | 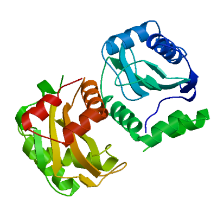 |
