# Supplementary material for: AI-2 quorum sensing-induced galactose metabolism activation in Streptococcus suis enhances capsular polysaccharide-associated virulence
Source: Vet Res. 2024 Jun 17;55:80. doi: 10.1186/s13567-024-01335-5 (PMC11184709; doi:10.1186/s13567-024-01335-5)
Supplement: Supplementary file 7 — Additional file 7. Docking results of AI-2 with FruA. [file 13567_2024_1335_MOESM7_ESM.docx]

**Additional file 7 Docking results of AI-2 with FruA.**

| Ligand | Receptor | Binding energy  (kcal·mol^-1^) | Binding affinity (kcal·mol^-1^) |
| --- | --- | --- | --- |
| AI-2 | FruA  (*S. suis*) | -2.72 | -5.1 |
|  | FruA  (*S pneumoniae*) | -2.40 | -4.8 |
